# 外泌体参与肺癌骨转移的机制研究及其相关临床诊疗进展

**DOI:** 10.3779/j.issn.1009-3419.2025.101.22

**Published:** 2025-11-20

**Authors:** YUAN Yuan, TANG Hao

**Affiliations:** 200003 上海，海军军医大学第二附属医院呼吸与危重症医学科; Department of Respiratory and Critical Care Medicine, Second Affiliated Hospital of Naval Medical University, Shanghai 200003, China

**Keywords:** 外泌体, 肺肿瘤, 骨转移, 诊疗, Exosomes, Lung neoplasms, Bone metastasis, Diagnosis and treatment

## Abstract

肺癌是发病率和死亡率最高的恶性肿瘤，诊断时通常已形成晚期转移。肿瘤发生转移是其治疗效果差、生存期短的重要影响因素，其中骨转移是肺癌常见的转移形式，常引起骨痛、病理性骨折、神经压迫和高钙血症等骨相关事件，严重影响患者生活质量且预后极差。外泌体是由细胞内多囊泡体与细胞膜融合后，释放到细胞外形成的直径为40-160 nm的细胞外囊泡。其携带蛋白质、脂质和核酸等物质介导肿瘤细胞与基质细胞、免疫细胞及骨微环境之间的信息传递，在肿瘤发生发展及侵袭转移过程中发挥重要调控作用，因此日益受到研究者们的关注。本文旨在总结并梳理外泌体促进肺癌骨转移的最新研究进展，重点阐述其关键机制与信号通路，包括在原发灶促进肿瘤细胞增殖和侵袭转移，在远处骨转移定植过程中参与的能量代谢重编程、转移前微环境塑造、免疫微环境调控和骨细胞、基质细胞等互作，并进一步分析外泌体在肺癌骨转移早期诊断、治疗及预后等方面的潜在临床转化价值。

## Abstract (en)

Lung cancer is the malignant tumor with the highest morbidity and mortality, and it is usually diagnosed with advanced metastasis. Tumor metastasis is an important factor affecting treatment resistance and poor prognosis of lung cancer, among which bone metastasis is a common metastatic pattern of lung cancer and is associated with a particularly dismal prognosis. Meanwhile, bone metastasis frequently causes skeletal-related events such as bone pain, pathological fractures, nerve compression, and hypercalcemia. Exosomes are extracellular vesicles with a diameter of 40-160 nm that are released from the fusion of intracellular multivesicular bodies and the cell membrane. Carrying proteins, lipids, and nucleic acids, they mediate communication between tumor cells and their surrounding matrix, immune cells, and the bone microenvironment. So, it has attracted increasing attention from researchers. This paper aims to summarize and organize the latest research advances on exosomes promoting lung cancer bone metastasis, focusing on their key mechanisms and signaling pathways. These include promoting tumor cells proliferation, invasion and metastasis at the primary lesion, as well as participating in energy metabolism reprogramming, pre-metastatic niche remodeling, immune microenvironment regulation, and interactions with osteocytes, stromal cells, and other cells during distant bone metastasis colonization. In addition, this review analyzes the potential clinical translational value of exosomes in the early diagnosis, treatment, and prognosis of lung cancer bone metastasis.

基于GLOBOCAN 2022的癌症统计数据^[1]^，肺癌占总体癌症死亡的18.7%并位居首位，严重威胁人类健康。在中国，肺癌同样是癌症相关死亡的主要原因之一，占总体癌症死亡的28.5%，成为全球肺癌负担最重的国家^[2]^。肺癌具有高复发和转移倾向，约40%的晚期肺癌患者会发生骨转移^[3]^。骨转移独特的骨痛、病理性骨折、神经压迫和高钙血症等骨相关事件（skeletal-related events, SREs）显著降低了患者的生活质量与治疗依从性，预后极差^[4]^。

目前，其临床治疗为化疗、靶向治疗等全身治疗，联合放疗、手术、骨水泥强化等局部干预的综合策略^[5,6]^。此外，抗骨吸收药物如唑来膦酸（zoledronic acid, ZA）与核因子-κB受体活化因子配体（receptor activator of nuclear factor-κB ligand, RANKL）抑制剂地舒单抗已被指南广泛用于预防和延缓SREs、减轻骨痛并改善骨相关结局，其中地舒单抗较ZA在延缓SREs方面更具优势^[7]^。然而，两者难以显著改善生存和逆转骨微环境重塑，均存在不良反应及成本等方面的限制，提示我们仍需从机制层面寻找更早、更准、更有效的诊疗突破口。此外，骨活检是诊断骨转移的金标准，确定其部位最广泛使用的技术以^18^F-氟代脱氧葡萄糖正电子发射断层显像/计算机断层扫描（fluorine-18 fluorodeoxyglucose positron emission tomography/computed tomography, ^18^F-FDG PET/CT）为核心，灵敏度和特异度优于CT和骨扫描等^[8-10]^。但其价格高昂，并且对于更早期的肿瘤细胞播散的检测和评估方面存在盲区，提示我们需要寻找其他简单、无创的辅助诊断方式。

外泌体作为直径为40-60 nm的细胞外囊泡，携带蛋白质、脂质和核酸等物质在体液中定向运输至靶组织并介导细胞间通讯^[11]^。自1983年被首次观察到并提出名称以来，Raposo等^[12]^证实其携带免疫分子主要组织相容性复合体II类分子并诱导抗原特异性T细胞表达，此后Valadi团队^[13]^进一步证明其可转运功能性mRNA和微小RNA（microRNA, miRNA），明确了外泌体在遗传信息转递与微环境重塑中的关键作用。

肿瘤来源外泌体可通过重塑骨微环境促进肺癌细胞转移，还可以影响转移灶细胞功能状态有助于细胞定植，成为潜在的诊断与干预靶标^[14]^。本综述将聚焦外泌体促进肺癌骨转移的分子与细胞机制，并总结其诊断与治疗的潜在价值，以期为肺癌骨转移的早诊早治与精准干预提供新思路。

## 1 外泌体参与肺癌骨转移

在肺内肿瘤原发病灶中，外泌体调控能量代谢、诱导上皮-间质转化（epithelial-mesenchymal transition, EMT）以及抑制免疫反应从而促进肺癌细胞生长转移；在骨转移灶形成的过程中，外泌体先于肿瘤细胞到达骨内并参与形成转移前生态位（pre-metastatic niche, PMN），同时调控肿瘤细胞的休眠与重激活并诱导破骨前体细胞大量分化形成溶骨性病灶，共同调控肺癌骨转移发生发展（图1A）。此外，部分外泌体表现出保护性作用。

**图 1:**
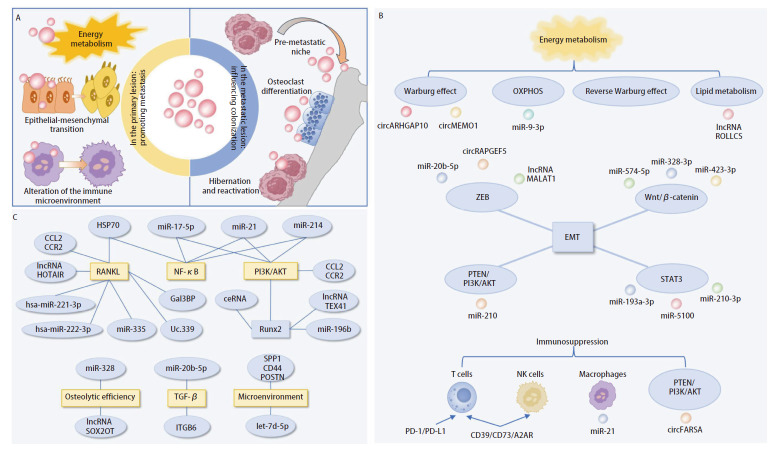
外泌体在肺癌骨转移中的关键作用概览。A：外泌体参与肺癌骨转移示意图；B：部分外泌体促进肺内肿瘤细胞生长和转移；C：外泌体影响肺癌细胞骨内定植。

### 1.1 外泌体影响肺癌细胞的生长和转移

外泌体通过调控肺内原发灶中肿瘤细胞的能量代谢和EMT，同时介导巨噬细胞极化等促进免疫抑制，从而影响肺内微环境以及改变肿瘤细胞的表型，促进肺癌细胞生长和远处转移（图1B）。

#### 1.1.1 能量代谢重编程

外泌体通过自分泌或旁分泌途径，向原发灶的肺癌细胞递送信号分子，进而实现快速供能满足其快速增殖、生长和远处转移的需要。有氧条件下，正常细胞更倾向于利用葡萄糖生成丙酮酸并进一步氧化磷酸化（oxidative phosphorylation, OXPHOS）供能，与糖酵解相比生成更多的三磷酸腺苷。与正常细胞相比，肿瘤细胞具有显著的代谢异质性。因此，代谢重编程被视为癌症的标志之一^[15]^。

外泌体驱动肿瘤细胞常常进行有氧糖酵解，即“Warburg效应”，表现为有氧环境下葡萄糖摄取升高及丙酮酸向乳酸转化增多，以快速供能维持生命活动。例如，血清来源的外泌体可增强肿瘤细胞中circARHGAP10的表达，靶向miR-638解除对下游通路的抑制，从而增强糖酵解，加速肿瘤细胞增殖和迁移^[16]^。此外，外泌体携带circ-MEMO1通过靶向miR-101-3p调节糖酵解代谢，促进肺癌的进展^[17]^。提示外泌体与环状RNA的信号通路是调节糖代谢的关键上游。

肿瘤细胞的代谢具有灵活性。外泌体还可在特定压力情况下增强线粒体的OXPHOS水平。研究^[18,19]^表明，外泌体携带miR-9-3p可以靶向醌氧化还原酶2（quinone oxidoreductase 2, NQO2）上调OXPHOS水平，通过增强线粒体功能促进肿瘤细胞持续生长。

外泌体还能在成纤维细胞、巨噬细胞等邻近细胞中诱导“反向Warburg效应”，为肿瘤细胞提供乳酸等代谢底物，从而提高OXPHOS水平^[20]^。与此同时，外排的乳酸形成的酸性微环境在肿瘤细胞外形成外酸内碱的pH梯度，降低细胞外基质（extracellular matrix, ECM）的力学约束并减少肿瘤细胞黏附，有利于其迁移与侵袭。

此外，部分外泌体携带长链非编码RNA（long noncoding RNA, lncRNA）ROLLCS等，利用脂质代谢增加能量供给和膜合成，从而提高促转移活性^[21,22]^。在肺癌细胞转移的过程中，肿瘤细胞的能量代谢根据不同环境和状态呈复杂的动态变化，这些作用共同促进原发灶的肿瘤细胞增殖与侵袭能力，为后续血行播散与疾病进展奠定代谢基础。

#### 1.1.2 诱导EMT

EMT是指细胞由“上皮样”向“间质样”状态转换，在肿瘤中表现为细胞极性和紧密连接减弱、上皮黏附分子下调以及侵袭迁移能力增强^[23]^。转移的过程中，外泌体作为重要的细胞间通讯载体，通过多条信号通路递送调控分子，诱导肿瘤细胞突破原发灶基底膜限制，持续推动EMT，从而为远处骨转移创造条件。

非小细胞肺癌（non-small cell lung cancer, NSCLC）来源的外泌体携带miR-20b-5p等微小核糖核酸调控转化生长因子-β（transforming growth factor-β, TGF-β）通路，增加锌指E盒结合同源框蛋白（zinc-finger E-box-binding homeobox, ZEB）等核心转录因子并降低上皮黏附分子的表达，同时靶向降低TGF-β受体II型（TGF-β receptor type II, TGFBR2）等蛋白水平促进黏附连接解体与细胞骨架重排，为EMT进程提供初始驱动信号，增强增殖、迁移、侵袭等恶性表型^[24,25]^。携带circRAPGEF5与lncRNA MALAT1的外泌体亦通过调控ZEB1巩固EMT并促进远处转移^[26,27]^。此外，miR-106a可被外泌体富集并跨细胞递送，通过调控肿瘤蛋白p53诱导的核蛋白1（tumour protein 53-induced nuclear protein 1, TP53INP1）放大EMT转录因子表达的同时下调自噬相关通路，促进肿瘤细胞播散^[28]^。

基质细胞来源的外泌体被肺癌细胞摄取后，可递送miR-210等分子，放大第10号染色体同源缺失性磷酸酶-张力蛋白基因（phosphatase and tensin homolog deleted on chromosome 10, PTEN）及磷脂酰肌醇3-激酶/蛋白激酶B（phosphatidylinositol-3-kinase/protein kinase B, PI3K/AKT）通路，进一步促进EMT与肿瘤细胞入血侵袭^[29]^。此外，骨髓来源间充质干细胞（bone marrow-derived mesenchymal stem cells, BMSCs）分泌的外泌体携带miR-574-5p、miR-328-3p及miR-423-3p等，靶向调控Wnt/β-连环蛋白（Wingless-type MMTV integration site family/β-catenin, Wnt/β-catenin）信号通路，放大EMT信号；miR-193a-3p、miR-210-3p及miR-5100则通过信号转导与转录激活因子3（signal transducer and activator of transcription 3, STAT3）信号通路进行EMT，从而促进肺癌骨转移^[30]^。

总的来说，外泌体首先激活TGF-β、PI3K/AKT等经典信号通路，进而调控EMT核心转录因子，最终通过影响自噬和蛋白翻译等过程为实现肺癌细胞EMT表型转化提供条件，提高其转移能力。

#### 1.1.3 形成抑制性免疫微环境

外泌体在肺癌原发灶及肺内微环境中通过多条免疫轴实现抑制性调控。

外泌体可以直接作用于T细胞的程序性细胞死亡受体1/配体1（programmed cell death protein 1/programmed cell death ligand 1, PD-1/PD-L1）通路，在引流淋巴结与外周循环范围内抑制T细胞活化；去除外泌体中PD-L1可恢复抗肿瘤免疫从而抑制肿瘤生长，血清外泌体中PD-L1水平与免疫治疗疗效有关，提示外泌体中PD-L1水平可作为动态免疫检查指标^[31]^。

外泌体还可以驱动腺苷信号轴建立代谢性免疫抑制。外泌体膜表达的核苷三磷酸二磷酸水解酶1（ectonucleoside triphosphate diphosphohydrolase 1, CD39）与胞外5’-核苷酸酶（ecto-5’-nucleotidase, CD73）将细胞外三磷酸腺苷转化为腺苷，从而经腺苷A2A受体（adenosine A2A receptor, A2AR）抑制效应T细胞与自然杀伤细胞的活性功能^[32,33]^。此外，针对CD39/CD73/A2AR通路的抑制剂与抗体正在与抗PD-1/PD-L1疗法联合使用，提示靶向外泌体中腺苷信号轴的转化潜力^[33]^。

肿瘤细胞外泌体还可以重塑先天免疫，尤其是参与巨噬细胞极化。肿瘤细胞可通过外泌体递送miRNA如miR-21等，直接激活Toll样受体/核因子-κB（Toll-like receptor/nuclear factor-κB, TLR/NF-κB）信号，诱导基质巨噬细胞向免疫抑制性M2型极化^[34,35]^。在基质中，成纤维细胞亦可通过外泌体携带的miR-3124-5p进一步放大此通路^[36]^。另一类通路是肿瘤来源外泌体携带环状核糖核酸circFARSA，靶向巨噬细胞PTEN及PI3K/AKT通路促进其极化，从而抑制免疫并提高肿瘤侵袭与播散能力^[37]^。

总之，外泌体通过上调PD-L1、调控腺苷信号轴或触发巨噬细胞M2极化共同降低免疫反应，为后续血行播散与远处骨转移提供免疫学前提。

### 1.2 外泌体驱动骨微环境重塑影响肺癌细胞骨内定植

肺癌骨转移的核心机制之一是外泌体对骨稳态的主动破坏。这些外泌体将特定的信号分子在转移前选择性富集于骨内，诱导PMN的形成，调控各细胞的休眠与重激活，继而驱动破骨细胞（osteoclasts, OC）分化和血管生成，形成有利于肺癌细胞骨内定植的微环境（图1C）。但仍有少数外泌体发挥抑制转移的保护性作用，外泌体在肺癌骨转移中的作用具有双重性。

#### 1.2.1 形成PMN

在肺癌细胞转移至骨内定植前，肿瘤来源外泌体可经由循环系统定向至骨内，诱导骨内基质细胞和免疫细胞的表型和功能改变，增强肿瘤细胞对骨的黏附与滞留，构成有利于肿瘤细胞着床的PMN^[38]^。

BMSCs可被肺癌来源的外泌体表面的热休克蛋白70（heat shock protein 70, HSP70）激活TLR2/NF-κB通路，使其向分泌型促炎间充质干细胞转化，上调RANKL/骨保护素（osteoprotegerin, OPG）比值，在转移前增强OC生成信号，为后续溶骨性病灶的发生奠定基础^[39]^。此外，外泌体通过C-C基序趋化因子配体2（C-C motif chemokine ligand 2, CCL2）/C-C基序趋化因子受体2（C-C motif chemokine receptor 2, CCR2）轴经循环系统到达骨内，激活骨内局部成纤维细胞等基质细胞分泌纤维连接蛋白，构建“着陆点”并完成骨转移灶的预改造；并进一步募集炎性单核细胞进入PMN，并驱动后者分化为免疫抑制性的巨噬细胞，有利于肿瘤细胞定向迁移和早期定植^[40]^。此后，外泌体通过CCL2/CCR2轴还可以促进肺癌细胞在骨内定植生长、并进一步通过RANKL信号通路促进OC分化。

巨噬细胞极化在PMN的形成过程中也发挥重要作用。携带circPLEKHM1的外泌体可诱导巨噬细胞向促转移表型极化和参与基质重塑^[41-43]^。外泌体还可摄取分泌型磷蛋白1（secreted phosphoprotein 1, SPP1）、细胞表面糖蛋白44（cluster of differentiation 44, CD44）和骨膜蛋白（periostin, POSTN）等物质，在促进肿瘤骨黏附的同时，形成支持肿瘤生长的骨微环境，进一步验证“种子与土壤”假说^[44]^。

此外，与原发灶内免疫抑制相似，外泌体可通过增强糖酵解或抑制骨转移灶内自然杀伤细胞和CD8^+^ T细胞等免疫细胞的抗肿瘤活性，建立免疫耐受和富含养分的微环境^[45]^。外泌体let-7d-5p被背根神经节神经元摄取后，通过靶向抑制μ型阿片受体1（μ-type opioid receptor 1, OPRM1），增加其对骨痛的敏感性以及通过释放神经肽激活骨微环境中的促炎因子，从而促进肿瘤细胞的存活和转移^[46]^。

总之，肺癌来源外泌体通过不同机制完成肿瘤细胞定植前的骨微环境塑造，同时也为开发靶向外泌体的抗转移治疗奠定基础。

#### 1.2.2 调控休眠与重激活

肺癌细胞转移至骨内定植后并非持续增殖，受机械刺激或化疗药物等影响，其可在休眠维持和重激活扩增之间动态切换，而外泌体是其中传递关键调控信号的重要媒介。

研究^[47]^表明，骨细胞可以通过感知运动产生的机械刺激，释放携带miR-99b-3p的外泌体。该外泌体被肺癌细胞摄取后，可抑制其增殖并维持休眠状态，从而延缓骨转移灶进展。相反，部分顺铂诱导的休眠的肺癌细胞并非完全静止，仍可分泌富含胰岛素样生长因子-2（insulin-like growth factor-2, IGF-2）和胰岛素样生长因子结合蛋白2（insulin-like growth factor binding protein 2, IGFBP2）的外泌体，这些外泌体被BMSCs摄取后，增强BMSCs中胰岛素样生长因子1受体（insulin-like growth factor-1 receptor, IGF-1R）介导的代谢重编程，从而促进糖酵解和乳酸分泌，降低阈值并形成有利于肿瘤细胞再生长与转移的骨髓生态位，从而使休眠的癌细胞可以被重新激活^[20]^。此外，外泌体整合素β6（integrin beta 6, ITGB6）可被成纤维细胞摄取，通过激活Kruppel样因子10（Kruppel-like factor 10, KLF10）正反馈环和TGF-β途径，诱导癌相关成纤维细胞活化并重建ECM，为肿瘤休眠后复发提供结构与信号基础^[48]^。

总之，外泌体在骨内既可传递抑制增殖信号以维持肿瘤细胞休眠，也可以通过重编程基质细胞代谢与ECM微环境降低阈值从而促进肿瘤细胞重激活，为探究骨转移灶潜伏期及治疗后复发提供机制线索。

#### 1.2.3 驱动OC分化

肺癌细胞来源的外泌体通过一系列功能相近的RNA和蛋白协调放大破骨信号，打破OC和成骨细胞（osteoblasts, OB）的平衡，促进溶骨性病灶的形成。

首先，外泌体携带多种RNAs如miR-21、miR-17-5p和miR-214等促进破骨信号通路。miR-21通过调控程序性细胞死亡蛋白4（programmed cell death protein 4, PDCD4）促进OC分化^[49]^。miR-17-5p则通过靶向负性调控分子PTEN，激活PI3K/AKT通路，上调骨吸收相关酶和基质降解蛋白表达，快速破坏骨小梁^[50]^。miR-214在增加OC前体向成熟OC分化的同时，还能通过抑制激活转录因子4（activating transcription factor 4, ATF4）抑制OB分化功能，从增强破骨和抑制成骨双向推动骨量丢失^[51]^。Runt同源盒转录因子2（Runt-related transcription factor 2, Runx2）作为调控骨代谢和肿瘤骨转移的关键转录因子，其上游同样受到外泌体竞争性内源RNA（competing endogenous RNA, ceRNA）网络的精细调控。在肺癌骨转移中共鉴定出2141个非编码核酸，显著富集于PI3K/AKT信号通路和TGF-β信号通路中介导肿瘤细胞与骨微环境之间的信号传递^[52,53]^。此外，外泌体携带lncRNA TEX41通过上调Runx2表达并增强保护性自噬；而miR-196b则通过靶向抑制Runx2调控PI3K/AKT信号强度，共同参与破骨相关转录程序的调控^[52,54]^。

其次，外泌体还通过放大RANKL通路与TGF-β/甲状旁腺激素相关蛋白（parathyroid hormone-related protein, pTHrP）信号，并破坏RANKL/OPG平衡，进一步加剧溶骨性失衡。lncRNA HOTAIR通过下调miR-138增强此信号，使OC前体在相对较低的RANKL水平下仍能高效分化^[55]^。半乳糖凝集素-3结合蛋白（galectin-3-binding protein, Gal3BP）^[56]^、外泌体超保守RNA339（ultra-conserved RNA 339, Uc.339）^[57]^、miR-335^[58]^亦增强RANKL信号，进一步促进OC前体高效分化。此外，外泌体携带hsa-miR-221-3p和hsa-miR-222-3p可直接结合并抑制雌激素受体1（estrogen receptor 1, ESR1），削弱后者对RANKL/OPG的保护作用，间接维持RANKL通路的优势；hsa-miR-151a-3p和hsa-miR-877-5p也参与肺癌骨转移^[53]^。多种miRNA通过不同靶点叠加激活上述经典通路，使破骨信号呈持续放大趋势。

除此之外，外泌体还可通过促进骨吸收小腔形成提升骨溶解效率。肺癌来源外泌体中miR-328可抑制神经纤毛蛋白-2（neuropilin-2, Nrp-2）表达，使OC前体中骨吸收相关基因上调，骨吸收小腔增多且体积增大，从而显著增强OC生成以及单位细胞的溶骨能力^[59]^。LncRNA SOX2OT则通过竞争性结合miR-194-5p，解除后者对Ras相关的C3肉毒素底物1（Ras-related C3 botulinum toxin substrate 1, RAC1）的抑制，使OC与骨表面的黏附更为牢固，提升骨基质局灶性溶解效率；同时也通过TGF-β/pTHrP/RANKL通路促进OC分化^[60]^。

#### 1.2.4 少数外泌体表现出保护性作用

有少部分外泌体表现出特殊的保护性作用。如外泌体携带miR-99b-3p以及上文提到的lncRNA TEX41等可通过上调Runx2表达并增强自噬在肺癌骨转移中起保护作用。外泌体miR-99b-3p和佐来膦酸联合治疗效果更佳^[47]^。这提示我们需要进一步探究外泌体在肿瘤进展中的作用机制。

总之，外泌体可以抑制负性调控因子、放大多个破骨信号以及提升溶骨效率共同推进肺癌细胞溶骨性转移灶的发生发展，亦可以通过少数非编码RNA发挥保护性作用，体现了外泌体在肺癌骨转移中的双重作用。

## 2 外泌体在肺癌骨转移中的临床诊断及治疗中的潜在价值

外泌体在肺癌骨转移中表现出双重作用。多数情况下，肿瘤来源外泌体促进骨微环境重塑、增强OC活化及促进肿瘤细胞骨内定植而推动骨转移进展；少数外泌体可向肿瘤细胞传递抑制增殖信号，维持休眠状态并延缓骨转移灶发展。基于外泌体的双向效应，其临床转化路径分为两个部分：阻断或抑制促转移的外泌体的发生、释放与受体细胞摄取；增强具有保护效应的外泌体或利用其货载的生物学特性作为药物递送平台从而精准干预骨转移。

外泌体包含多种有效的功能分子，通过调控OC分化、驱动EMT、PMN、免疫逃逸以及调控能量代谢和休眠与重激活等机制，参与肺癌骨转移进展。这些机制的明确为辅助临床诊断及治疗提供潜在价值。外泌体来源特异且稳定存在于血清等体液中，其可动态反映肿瘤负荷与微环境状态，具有作为特异性液体活检标志物的潜力。当前研究主要提示其可作为影像学与骨代谢指标之外的补充辅助诊断，用于骨转移风险预测或早期提示。此外，随治疗过程动态检测外泌体的变化，有利于辅助判断骨转移灶活动度、潜在复发风险与患者预后。为此，我们将相关促转移和抑制转移的外泌体及其相关靶点在临床中的潜在价值及研究阶段绘制成表格以供参考，见表1^[28,30,39,40,42,44,46,47,50,51,53,55-58,61]^。

**表 1:** 外泌体及其相关靶点在肺癌骨转移中所处的研究阶段及其潜在临床价值

Exosomes and their related targets	Potential clinical value	The current research stage
miR-106a, TP53INP1^[28]^	Potential therapeutic targets for bone metastasis of lung adenocarcinoma	Clinical samples and mechanism research
miR-574-5p, miR-328-3p, miR-423-3p^[30]^	Diagnostic markers for bone metastasis of lung cancer	Clinical samples
miR-21^[61]^	Targeting the potential targets of osteoclastic differentiation in bone metastasis of lung cancer	Clinical samples
HSP70^[39]^	Potential diagnostic markers for bone metastasis of lung cancer	Animal experiment
CCL2/CCR2^[40]^	Prognostic markers and therapeutic targets for NSCLC metastasis	Animal experiment
circPLEKHM1^[42]^	Metastasis and prognostic markers of NSCLC	Animal experiments and clinical samples
SPP1, CD44, POSTN^[44]^	Predicting the risk of bone metastasis from lung cancer	Clinical samples
let-7d-5p^[46]^	Potential targets for the prevention and treatment of cancer-induced bone pain	Animal experiment
miR-17-5p^[50]^	Therapeutic targets for bone metastasis of NSCLC	In vitro experiment
miR-214^[51]^	Risk of bone metastasis from lung cancer, potential preventive and therapeutic targets	Clinical samples
lncRNA HOTAIR^[55]^	Prognostic markers and potential therapeutic targets for bone metastasis of lung cancer	In vitro experiment
Gal3BP^[56]^	Prognostic markers and potential therapeutic targets for bone metastasis of lung cancer	In vitro experiment
Uc.339^[57]^	Potential therapeutic targets for bone metastasis of lung adenocarcinoma	Animal experiment
miR-335^[58]^	Potential diagnostic markers for bone metastasis of lung cancer	Animal experiment
hsa-miR-151a-3p, hsa-miR-877-5p^[53]^	Potential diagnostic markers for bone metastasis of lung cancer	Clinical cohort study
miR-99b-3p^[47]^	Potential therapeutic targets for bone metastasis of lung cancer, combined therapy with zoledronic acid	Clinical samples

HSP70: heat shock protein 70; NSCLC: non-small cell lung cancer; lncRNA: long noncoding RNA.

此外，部分药物如nSMase相关通路抑制剂GW4869等可以干预外泌体携带或释放物质出胞的过程，减轻溶骨性破坏并改善治疗效果。我们也可以利用外泌体天然递送与组织趋向特性，利用工程化外泌体作为药物载体定向递送至肿瘤部位发挥治疗作用，见表2^[62-66]^。

**表 2:** 抑制促转移外泌体释放或利用工程化外泌体参与肺癌骨转移治疗

Therapeutic measures	Specific approaches	Effect
GW4869^[62]^	Used alone or in combination with first-line chemotherapy	Reduce chemotherapy tolerance and distant metastasis
siRNA, CRISPR knock down Rab27A/Rab27B, Nexinhib20^[63]^	Inhibit the key enzymes of the exosome release pathway or interfere with their interaction with effector proteins	Reduce the exocytosis and growth metastasis of exosomes
LincRNA01703^[64]^	Enhance the function of the Rab27a/SYTL1/CD81 complex	Inhibit the infiltration and distant metastasis of immune cells
Mesenchymal stem cells or engineered tumor cell exosomes^[65]^	Delivery of anti-KRAS/EGFR siRNA Small molecule drugs or anti-cancer miRNAs	Inhibit the growth of the primary lesion and metastatic lesions and improve targeting or chemotherapy sensitivity
Engineered small extracellular vesicles^[66]^	Delivering drugs and surface-modifying bones towards receptors to reduce osteolysis	Reduce osteolysis and bone pain

Rab27a/SYTL1/CD81: Ras-related protein Rab-27A/synaptotagmin-like protein 1/cluster of differentiation 81; KRAS/EGFR: Kirsten rat sarcoma viral oncogene homolog/epidermal growth factor receptor.

目前专门针对肺癌骨转移的前瞻性临床研究仍不足。未来需要我们在统一外泌体分离检测标准的基础上，结合前期已有机制研究进展，设计针对骨转移高危人群的验证队列和早期干预试验，真正推动外泌体从机制走向临床，提高患者生活质量。

## 3 总结与展望

现有的研究表明，肺癌来源外泌体通过递送多种非编码RNA和蛋白质，协同调控原发灶肿瘤细胞恶性进展与EMT，同时在远处骨内诱导PMN形成以及OC活化，贯穿整个肺癌骨转移的发生发展。对其机制研究的不断深入有助于为临床诊断标志物的发现和治疗靶点提供分子基础。血清来源的外泌体与肺癌骨转移密切相关，可用于无创辅助筛查、辅助早期诊断、疗效检测和预后分析。其中，大多数的外泌体携带致癌分子，促进肿瘤转移，但仍有少数表现为缓解癌症的保护性作用。

从基础研究到真正临床应用仍面临多重挑战。在基础研究方面，现有的大多数研究聚焦于肺腺癌等NSCLC中，在小细胞肺癌及罕见亚型中的研究不足。其次，机制研究在细胞层面以OC为核心，对OB、骨细胞、基质细胞及神经元等发挥的作用的研究尚不充分。此外，目前多数研究以回顾性、小样本或单中心为主，尚缺乏基于标准化平台的大规模多中心前瞻性队列。

在技术层面，外泌体的高度异质性导致尚未完全明确在肺癌骨转移中的亚型，现有的外泌体分离纯化技术各有优缺点，仍处于发展的早期，不同实验室流程差异较大、缺乏结果可比性仍是瓶颈问题^[67]^。另一方面，外泌体在其他疾病中的机制研究及初步临床探索，为肺癌骨转移领域提供重要思路。

随着技术的进步和研究的深入，外泌体有望成为肺癌骨转移早期诊断、预后评估和治疗的新工具，为改善患者预后提供新思路，以期于在未来外泌体能联合PET/CT、骨扫描及穿刺的多模态综合评估，达到早诊、优治的临床效果。相信在未来，外泌体在基础机制、技术优化和临床转化三个维度协同推进，可以实现从实验室到临床的真正突破，进而使更多的肺癌骨转移患者从中获益。
